# Functional Nutrition as Integrated Intervention for In- and Outpatient with Schizophrenia

**DOI:** 10.2174/1570159X21666230322160259

**Published:** 2023-09-25

**Authors:** Francesco Matrisciano

**Affiliations:** 1 The Psychiatric Institute, Department of Psychiatry, College of Medicine, University of Illinois Chicago (UIC), Chicago, IL, USA

**Keywords:** Schizophrenia, neuroinflammation, synaptic plasticity, nutrition, functional foods, bioactive compounds

## Abstract

Schizophrenia is a chronic and progressive disorder characterized by cognitive, emotional, and behavioral abnormalities associated with neuronal development and synaptic plasticity alterations. Genetic and epigenetic abnormalities in cortical parvalbumin-positive GABAergic interneurons and consequent alterations in glutamate-mediated excitatory neurotransmission during early neurodevelopment underlie schizophrenia manifestation and progression. Also, epigenetic alterations during pregnancy or early phases of postnatal life are associated with schizophrenia vulnerability and inflammatory processes, which are at the basis of brain pathology and a higher risk of comorbidities, including cardiovascular diseases and metabolic syndrome. In addition, schizophrenia patients adopt an unhealthy lifestyle and poor nutrition, leading to premature death. Here, I explored the role of functional nutrition as an integrated intervention for the long-term management of patients with schizophrenia. Several natural bioactive compounds in plant-based whole foods, including flavonoids, phytonutrients, vitamins, fatty acids, and minerals, modulate brain functioning by targeting neuroinflammation and improving cognitive decline. Although further clinical studies are needed, a functional diet rich in natural bioactive compounds might be effective in synergism with standard treatments to improve schizophrenia symptoms and reduce the risk of comorbidities.

## 1 CLINICAL CHARACTERISTICS AND IMPLICATIONS OF SCHIZOPHRENIA

Schizophrenia is a severe chronic neurodevelopmental disorder associated with progressive neuronal loss of structure and function [1, 2] and characterized by late adolescence or early adulthood clinical manifestation, although the neuropathology begins during prenatal development [3-5]. Despite its relatively low prevalence (the global age-standardized point prevalence of schizophrenia in 2016 was estimated to be 0.28%) [6], its prevalence varies amongst specific subgroups and high-risk populations [7]. As described in the Diagnostic and Statistical Manual of Mental Disorders, Fifth Edition (DSM-5), the main schizophrenia symptoms are delusions and hallucinations, disorganized thinking and speech, negative symptoms such as affect flattening and avolition, and cognitive impairment, such as alterations in selective attention, working memory, executive function, episodic memory, language comprehension, and socio-emotional processing skills, which represent the core domain of the disease and the best predictor of long-term functional outcome [8, 9]. Risk factors associated with schizophrenia include maternal stress, maternal infections and maternal nutritional deficiencies that affect fetal neurodevelopment, intrauterine growth retardation, pregnancy, and birth complications, as well as socioeconomic factors and early life adverse experiences [10-12]. The cognitive impairment associated with schizophrenia (CIAS), a core feature of schizophrenia, is caused by alterations in GABAergic/glutamatergic neurotransmission in the frontal lobe and constitutes the most resistant disease domain to antipsychotic treatment [13-16]. Moreover, schizophrenia patients show a higher risk of premature death by all causes due to comorbid medical conditions associated with socioeconomic and lifestyle factors, including nutrition status and sedentary behavior, which are modifiable risk factors for cardiovascular diseases, insulin resistance, and metabolic syndrome [17-20]. Premature death results from poor physical health outcomes, especially in the outpatient setting with lack of care. In fact, life expectancy for individuals with schizophrenia has been estimated to be shorter than the general population because of higher mortality risk due, mainly, to tobacco and alcohol abuse, cardiovascular diseases, poor diet, physical inactivity, and metabolic syndrome [21]. Thus, an integrated nutritional approach is needed to improve the disease course and prevent comorbidities.

Despite the benefits induced by second-generation antipsychotics, particularly long-acting formulations in terms of efficacy, tolerability, and quality of life [22, 23], additional strategies to strengthen functional recovery (*e.g*., recovery of the capacity to maintain employment or self-care independency) need largely to focus on addressing general health conditions and cognitive deficits, which remain still an unmet goal and require urgent attention.

This review aims to address the role of functional foods as a natural potential tool to improve the overall health and quality of life of individuals with schizophrenia by targeting systemic and brain inflammation as the molecular mechanism underlying neuropathology.

## 2 SCHIZOPHRENIA: PATHOGENETIC MECHANISMS

The causes of schizophrenia are not completely understood. Besides genetic vulnerability, recent findings strongly support the role of altered epigenetic processes that result from an abnormal interplay between individual genetic susceptibility and early-life stressful events, causing alterations in neurotransmission at the cortical level [24-27]. GABAergic interneurons play a fundamental role in orchestrating the critical imbalance between the inhibitory neurotransmission driven by GABA and excitatory neurotransmission mediated by glutamate [28-34]. Preclinical studies showed epigenetic and expression changes in glutamate decarboxylase (GAD), reelin and brain-derived neurotrophic factor (BDNF) associated with increased levels of DNA methyltransferases (DNMT) in GABAergic interneurons strongly supporting the hypothesis that schizophrenia represents an “interneuronopathy” in which early onset epigenetic changes in GABAergic interneurons in the frontal cortex cause a disruption in GABAergic/glutamatergic neurotransmission and abnormalities in the oscillation network [35-40]. Glutamate is also involved in tryptophan metabolism and the kynurenine pathway, which is also stimulated by pro-inflammatory cytokines, and the neuroactive metabolite kynurenic acid acts, in turn, as an NMDA receptors antagonist linking the glutamate hypothesis of schizophrenia to inflammation and glia activation [41, 42]. Neuroinflammation mechanisms in schizophrenia have been recently investigated and supported as relevant for its pathogenesis, as demonstrated by several ongoing clinical trial studies focused on immune-inflammatory targets as potential treatments for schizophrenia (clinicaltrial.gov/ inflammation-schizophrenia) [43-47]. Individuals with schizophrenia show elevated expression of inflammatory markers such as interleukin (IL)-β, IL-6 or C-reactive protein (CRP) in both brain and peripheral blood, thus targeting the neuro-immune system with immune-modulators, anti-inflammatory drugs, antioxidants, nutrients, vitamins, and neuroprotective agents is currently under consideration for the disease management [47-49]. Based on the “second hit” hypothesis of the disorder manifestation and progression, adverse events or “stressors, ” including trauma, physical or psychological abuse or use of drug abuse occurring during puberty or early adulthood in individuals with strong genetic vulnerability, lead to symptoms onset, which is also referred as “first episode” psychosis (FEP) [50]. After the first episode, the disease shows a relapse-remitting pattern, and it has been reported that a certain percentage of individuals do not respond to either second-generation, first-choice antipsychotics, or clozapine used as the second choice for its potential adverse events and tolerability profile and indicated for schizophrenia-resistant patients [51]. For clozapine-resistant patients, defined as “ultra-resistant” individuals, different pathological mechanisms, including neuroinflammation and oxidative stress, have been observed in association with worsening outcomes, including functional disability and death [52].

### 2.1 Role of Inflammation in Schizophrenia

The mechanism that links schizophrenia to inflammation relies on the evidence that systemic inflammation caused by different pathological conditions leads to pro-inflammatory cytokines release into the general system, which then creates a mirror inflammatory response in the brain *via* microglia activation and secondary production of pro-inflammatory cytokines, such as TNF, IL-1β and IL-6 [40].

Chronic low-grade systemic inflammation and consequent activation of neuroinflammatory processes through reactive microglia in the brain represent a well-known pathogenetic mechanism for schizophrenia [53-55]. Peripheral inflammatory stimuli reach the brain and trigger astrocytes and microglia activation [56, 57]. Microglia cells constitute the innate immune cells and, in the absence of inflammation, are generally in a surveillant state [58, 59]. Moreover, triggered astrocytes can transform into a pro-inflammatory phenotype and regulate nuclear factor kappa-light-chain-enhancer of activated B cells (NF-κB) expression and the production of pro-inflammatory cytokines through toll-like receptor (TLR)4, which then stimulate microglia activation.

The transcription factor NF-κB is a key molecular player for immune response regulation and mediates the expression and release of various pro-inflammatory cytokines during inflammatory processes. The brain of individuals with schizophrenia showed increased expression of the pro-inflammatory state that correlates with the severity of symptoms [60, 61]. It has also been suggested that NF-κB is overactive in the cortex in people with schizophrenia and drives neuroinflammation due to decreased expression of the NF-κB inhibitor, human immunodeficiency virus type 1 enhancer binding protein 2 (HIVEP2) [62]. Although overactivation of NF-κB has been considered one of the mechanisms involved in the pro-inflammatory state of the brain of schizophrenia patients [63], a strict correlation between NF-κB expression and neuroinflammation in schizophrenia patients is difficult to establish due to the high complexity non-canonical and canonical upstream activators of NF-κB and its regulation. Higher expression of NF-κB is also present in individuals with neuroinflammation regardless of diagnosis [64].

Moreover, microglia-neuron crosstalk *via* pro-inflammatory molecular players leads to neuroinflammation and neurodegeneration, causing alterations in synaptic plasticity and neuronal death, affecting the main mechanisms underlying learning and memory processes [60, 61]. Microglia play an active role in defending the central nervous system through surveillance activity and responding to challenging events that cause damage to neuronal cells [62]. This occurs through activating a cascade of inflammatory processes mediated by TLR signalling that converges to the NF-κB transcription factor [63]. A recent study showed increased levels of TNF-α and IL-6 were associated with a subgroup of the population with chronic schizophrenia. Inflammation processes lead to increased oxidative stress and the consequent cell damage and neurodegeneration [65].

In addition, alcohol, drugs of abuse, stress, and infections, common among schizophrenia populations, activate neuroinflammatory responses. Abnormal expression of TLR has been reported in the early stages of schizophrenia and is linked to cognitive deficits. Moreover, systemic inflammation can also affect the brain and its development [64-66].

Abnormal activation of neuroinflammatory mechanisms is strongly associated with schizophrenia severity and progression. Thus, prevention of anti-inflammatory processes, including adopting nutritional interventions with anti-inflammatory bioactive compounds, might lower the impact of inflammation and oxidative stress on brain functioning and may reduce (i) the illness severity and progression, (ii) the adverse metabolic effects induced by antipsychotics, and (iii) the risk of comorbidities.

## 3 CURRENT TREATMENTS FOR SCHIZOPHRENIA: UNMET NEEDS

Although a full pharmacological description of current treatments for schizophrenia is not the purpose of this manuscript, current clinical interventions for schizophrenia management include, amongst others, (i) first- (haloperidol) and second-generation antipsychotics, partial agonists, oral or long-acting formulations, which are the commonly prescribed drugs to treat psychotic disorders including schizophrenia; (ii) psychological and psychosocial interventions; (iii) primary health care. First-generation antipsychotics (FGAs) and second-generation antipsychotics (SGAs) differ mainly in their pharmacological profile, tolerability, and risk of side effects, with SGAs associated with a higher risk of metabolic syndrome (obesity, hypertension, diabetes, dyslipidemia). It is well-established that individuals with schizophrenia are affected by the cardiometabolic, endocrine, and motor adverse effects of current antipsychotic medications, putting these individuals at increased risk of morbidity and mortality [68]. Although with the development of the “partial agonists for D2 and D3 receptors” such as aripiprazole and cariprazine, the tolerability and risk of metabolic side effects have been addressed, yet, finding additional pharmacological and non-pharmacological strategies to address this matter is becoming urgent and fundamental due to suboptimal outcomes of most patients and the lack of data or observations on their role in neuroinflammation.

Pharmacological treatment of schizophrenia relies on second-generation antipsychotic drugs, oral or long-acting formulations, such as the chemical structure-related clozapine, olanzapine, or quetiapine, aripiprazole, risperidone, paliperidone and lurasidone. They all mainly target, with different affinity, specific dopamine and serotonin receptor subtypes [67]. Lumateperone, the novel antipsychotic drug that recently received its first global approval in the USA, acts as a modulator of serotonin, dopamine, and glutamate [69], supporting the role of glutamate in schizophrenia. A recent study showed that lumateperone demonstrated efficacy in improving the symptoms of schizophrenia and had a favorable safety profile.

Antipsychotic drugs generally show an optimal efficacy profile for positive symptoms but relatively poor efficacy for negative or cognitive symptoms whose treatment remains an unmet goal [69]. Schizophrenia management is also complicated by the fact that antipsychotic treatment usually begins after the onset of clinical manifestation (first episode of psychosis or FEP), which appears years after the neuropathology begins, creating an untreated gap between the time of brain dysfunction and symptoms manifestation [31]. In addition, the tolerability profile can be challenging in many cases because of adverse effects, such as sedation, weight gain, metabolic syndrome, and anticholinergic side effects, which worsen cognitive impairment at a relatively young age [70]. Individuals with schizophrenia also face the disease's impact on their quality of life. Most early functional disability cases and early deaths of individuals with schizophrenia are attributable to preventable medical conditions, including poor health care, cardiovascular diseases, obesity, diabetes, dementia, and tobacco-induced diseases, such as chronic obstructive pulmonary disease (COPD) and some types of cancer [71]. Thus, considering novel integrated strategies, including non-pharmacological interventions, is strongly recommended for improving long-term outcomes and quality of life for those living with schizophrenia.

Recently, for schizophrenia pathogenesis, the focus has been given to the role of systemic low-grade chronic inflammation and neuroinflammation and the impact of nutrition on these processes [72]. Chronic low-grade systemic inflammation, characterized by hyper-production and activity of cytokines with secondary neurotoxic effects, is involved in developing mental illnesses, including schizophrenia. Although low-grade systemic inflammation and neuroinflammation represent well-established risk factors for schizophrenia pathogenesis, the activation causes are less understood. Psychological stress, smoking, obesity, physical inactivity, substance use and alcohol use, lack of sleep and poor diet may play a relevant role in igniting inflammation [73, 74]. This also suggests that nutrition and bad eating habits are associated with the severity of symptoms and disease progression. It is well-established that high-calorie diets promote oxidative stress and chronic systemic low-grade inflammation that predispose to brain dysfunction and neurodegeneration in the hippocampus, a brain region that regulates important functions, such as working memory and executive functions [75-77].

## 4 NOVEL MOLECULAR TARGETS FOR SCHIZOPHRENIA

Schizophrenia is a severe and complex disease characterized by high heterogeneity in clinical manifestations and neuropathological mechanisms that requires a broad therapeutical approach finalized not only to targeting the alterations in neurotransmission but also to correcting the neuronal damage and cognitive decline induced by abnormalities in GABAergic interneurons/glia/neurons crosstalk within the cerebral cortex linked to immune-inflammatory-induced cytotoxic effects.

The main purpose of this section is to bring to light novel potential molecular targets that may lead to studies that examine their role in novel interventional strategies. Adopting functional nutrition rich in polyphenols with anti-inflammatory effects, including quercetin, curcumin, resveratrol, and lycopene, amongst others, might support the creation of more structured preclinical and clinical research studies that address the main outcome, not just the control of psychotic symptoms but also the overall health, the risk of comorbidity and mortality induced by underlying chronic inflammatory processes associated with schizophrenia.

Alterations in several neurotransmitters in different brain areas are strongly involved in schizophrenia pathogenesis. Cortical and subcortical dopamine dysfunction has been considered for decades the “core” of the pathogenesis of schizophrenia (dopamine hypothesis), specifically for the development of psychotic and positive symptoms associated with a striatal increase in pre-synaptic dopamine synthesis and release while reduced dopamine neurotransmission in cerebral cortex linked to negative and cognitive symptoms of schizophrenia. The dopamine hypothesis led to the development the dopamine D2 receptor-centered neuroleptics whose prototype is represented by haloperidol. More recently, several studies unveiled the role of alterations in other neurotransmitters such as serotonin, glutamate, GABA and acetylcholine [78], which led to the discovery of the role of the 5-HT2A receptor antagonism in the antipsychotic activity of second-generation antipsychotics.

From a clinical perspective, pharmacological treatment of schizophrenia relies on a broad range of molecules belonging to first-generation antipsychotics (haloperidol, chlorpromazine), second-generation antipsychotics (clozapine, olanzapine, risperidone, quetiapine), dopaminergic partial modulators (aripiprazole, brexpiprazole, cariprazine), serotonergic/ dopaminergic/ glutamatergic antipsychotic (lumateperone), and muscarinic cholinergic receptor ligands (xanomeline) amongst others including long-acting injectable antipsychotics. Despite this multitude of molecules, the pharmacological treatment of schizophrenia still shows profound limitations, which include: i) poor or lack of efficacy for negative and cognitive symptoms; ii) side effects including metabolic syndrome (dyslipidemia, hypertension, diabetes and obesity), which is sadly linked to a higher mortality rate of schizophrenia population due to medical conditions, mostly cardiovascular diseases; iii) lack of response of individuals with characteristics of treatment resistance (30% of patients do not respond to clozapine (ultra-resistant patients); iv) risk of relapse and consequently use, despite the evidence of little or no efficacy, of two or more antipsychotics in combination with consequent increased risk of side effects. Thus, a broader approach to treating and managing these individuals is needed to ameliorate their quality of life and improve their life expectancy. The World Health Organization (WHO) defines health as a condition of complete physical, mental, and social well-being and not merely the absence of disease or infirmity. Also, it defines the determinants of health, such as lifestyle factors, including physical activity and diet. Improving the unhealthy lifestyles of individuals with severe mental disorders such as schizophrenia, bipolar, and major depression in a home- or community setting must be considered an essential part of treatment by implementing psychosocial and educational programs.

Addressing the underlying inflammatory processes and metabolic disorders associated with chronic schizophrenia may represent an integrated approach to improving the quality of life of these individuals. Recently, Keller *et al.* (2018) reported that treatment strategies using anti-inflammatory agents showed some benefits in people with schizophrenia [79]. Moreover, abnormalities in the gut system that trigger inflammation are linked to schizophrenia and interventions such as dietary modifications may help to reduce symptom severity [80].

Pro-inflammatory markers, such as NFκB, PGE2, iNOS, and COX-2, are highly expressed in a cohort of schizophrenia patients compared to healthy controls [81]. Thus, we can speculate that targeting these molecular players might represent a suitable therapeutic candidate for schizophrenia treatment. Moreover, it is well-established that peroxisome-proliferator-activated receptors (PPARs) exert anti-inflammatory effects by inhibiting the NF-κB pathway [82]. In the schizophrenia animal model of maternal immune activation (MIA), prenatal treatment with the PPAR-α agonist, fenofibrate, attenuates the MIA-induced biochemical and behavioral deficits [83]. Favourable effects were also observed in managing the second-generation antipsychotic olanzapine-induced weight gain by using a histamine agonist with PPAR-α modulating activity [84, 85]. A recent randomized, double-blinded, placebo-controlled trial studied the efficacy and tolerability of palmitoylethanolamide (PEA) in treating negative symptoms in patients with stable schizophrenia. This 8-week trial found that combined therapy with PEA and risperidone safely alleviates schizophrenia-related primary negative symptoms [86].

In line with this evidence, one of our recent studies showed that in a mouse model of social isolation, stress-induced epigenetic (hypermethylation) changes in PPAR-α expression with consequent alterations in neurosteroid synthesis and emotional behavior dysfunction, suggesting that i) PPAR-α might be a suitable candidate for potential further clinical investigations, and ii) the stimulation of PPAR-α induced by PEA might reverse the epigenetic changes and ameliorate the pathological phenotype [87].

Neuroinflammation during development or early life, in turn, alters neuronal maturation and synaptic plasticity by altering the glutamatergic system [88]. Through microglia-neuron crosstalk, pro-inflammatory cytokines, including TNF-α, alter the cell membrane expression of AMPA and NMDA glutamate receptors, leading to abnormal calcium mobilization and precipitate excitotoxicity processes. They also inhibit glutamate transport on astrocytes and alter GABAA receptor expressions [85]. Several food-derived bioactive compounds and phytonutrients act as anti-inflammatory agents targeting several molecular players involved in inflammation, including the PPAR-α, polyphenols, and unsaturated fatty acids that stimulate its expression [89].

**Fig. (1):**
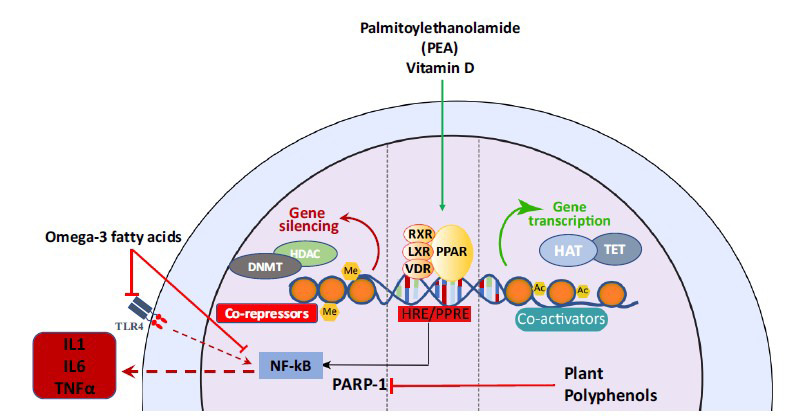
A simplified representation of molecular targets involved in inflammatory processes within the brain. Stimulation of PPAR/RXR complex by PEA or vitamin D induce epigenomic effects leading to activation of gene transcription and anti-inflammatory effects *via* inhibiting NF-kB signalling. Plant polyphenols including flavonoids and isoflavonoids including EGCG, resveratrol, apigenin inhibit PARP-1 with consequent anti-inflammatory effects by blocking NF-kB stimulation and decrease in pro-inflammatory cytokines release. Similarly, omega-3 fatty acids inhibit NF-kB/TLR4 pro-inflammatory signalling promoting anti-inflammatory effects.

Recently, another potential molecular mechanism involves the poly (ADP-ribose) polymerase (PARP), a DNA repair enzyme and transcription factor which regulates neuroinflammation by stimulating NF-kB [90]. Several natural polyphenols have been identified with inhibitory effects on PARP, including flavonols such as quercetin found in cranberries, raspberries, blueberries, and onions, as well as in buckwheat, tea, red wine, and olive oil [91]. So, PARP inhibitors may be suitable candidates for inflammatory-based neuropsychiatric disorders, including schizophrenia. PARPs, a family of nuclear proteins involved in several physiological and pathological cellular processes, including inflammation and DNA repair thanks to their capability of DNA-binding and enzymatic activity [92], has emerged as a therapeutic target in cancer treatment, including ovary and breast, and neurodegenerative disorders [93, 94]. It has been reported that hyperactivation of PARP-1 plays an important role in the development of diseases directly or indirectly associated with chronic inflammation, including diabetes, neurodegenerative disorders (Alzheimer’s disease (AD), Parkinson’s disease (PD)), and cardiovascular diseases [95] (Fig. **1**). A recent study reported that mice with PARP-1 deficiency showed deficits in neurogenesis during development and adulthood phases with brain regional differences associated with abnormal behaviors resembling schizophrenia-like behavioral abnormalities [96], suggesting that further studies are needed to clarify the PARP-1 role in schizophrenia phenotype.

## 5 BIOACTIVE COMPOUNDS FOR INTERVENTIONAL STRATEGY IN SCHIZOPHRENIA

Table **1** shows a list of bioactive compounds that may demonstrate potential benefits for schizophrenia long-term management by impacting the immune-inflammatory processes. Several foods rich in bioactive compounds such as omega-3 polyunsaturated fatty acids, flavonoids, phytonutrients, minerals, and vitamins showed beneficial effects in psychosis [76]. Adopting a diet rich in bioactive compounds active against oxidation and inflammation might improve the overall health and quality of life of schizophrenia patients by decreasing the risk of comorbidities and reducing neuroinflammation.

Table **2** reports some clinical studies showing the physiological effects of bioactive compounds in schizophrenia and metabolic syndrome.

Here, we describe the biological activity of some of the natural compounds that target schizophrenia pathogenesis and improve illness severity and progressiveness by targeting neuroinflammation or metabolic syndrome.

### 5.1 Role of Vitamins in Schizophrenia

Poor prenatal and postnatal nutrition, vitamin deficiencies, and foods lacking anti-inflammatory and antioxidant activity are considered risk factors for schizophrenia [94-98]. A recent study has described eating and nutritional habits in patients with schizophrenia, reporting that the clinical severity of the illness was associated with poor eating habits and nutrient deficit [99]. Vitamins A and D are lipid-base compounds and activate nuclear receptors inducing genomic effects. Vitamin A (retinoic acid or RA), which derives mostly from dietary sources such as carrots and sweet potatoes, has been implicated in different biological functions, including cognitive deficits in Alzheimer’s disease and schizophrenia [100].

Its biological effects also relate to its ability to activate nuclear receptors, including PPARs and regulate the expression of target genes. In fact, the retinoid “X” receptors (RXRs) form heterodimers with many other nuclear receptors, such as the thyroid hormone receptor (RXR-TR), the Vitamin D3 receptor (RXR-VDR), the PPAR (RXR-PPAR) and the liver “X” receptor (RXR-LXR) [101]. The ligand-activated complex then binds to responsive elements on DNA and regulates the transcription of a subset of genes involved in development and cell functions, including differentiation, energy metabolism, insulin sensitivity and inflammation, by inducing changes in chromatin structure [101, 102]. Altered RA signaling leads to neuroinflammation, oxidative stress, mitochondrial malfunction, and neurodegeneration processes, which are also involved in schizophrenia pathogenesis.

Another lipid-base neuroactive vitamin is vitamin D. Vitamin D, a fat-soluble vitamin along with vitamins A, E and K, is a steroid hormone mostly known for its role in calcium metabolism and its ability to increase the absorption of calcium and phosphorus from the intestine, but vitamin D exerts many other biological effects including processes involved in brain development and neuronal activity [103]. Also, vitamin D regulates serotonin synthesis by transcriptional activin of the enzyme tryptophan hydroxylase 2 and dopamine release [104-107].

Alterations in vitamin D levels are associated with immune system dysregulation, systemic inflammation and neuroinflammation. Vitamin D receptor (VDR) belongs to the nuclear receptors family expressed in both glia and neurons and can dimerise with retinoic X receptors (RXRs) to regulate gene transcription. Deficiency in vitamin D is considered a risk factor for neuropsychiatric disorders, such as schizophrenia and cognitive impairment in adults, causing alterations in brain structure and dopamine and glutamate signalling [108]. Many studies have focused on the role of vitamin D in schizophrenia patients by measuring its plasma levels. Low vitamin D during the early phases of life has been reported to be associated with schizophrenia [109]. Vitamin E also belongs to the fat-soluble vitamins, and α-tocopherol is linked to genomic effects targeting inflammation, cell signalling, and cell cycle regulation [110]. Vitamin E regulates gene expression through its interaction with α-tocopherol transfer protein (α -TTP) and secondarily transfers into the cell and binding to the “antioxidant responsive element” (ARE) or *via* liver X receptor (LXR) to activate specific gene targets involved in inflammation. Clinical studies showed a deficiency of α-tocopherol in schizophrenic patients compared to controls [111].

Vitamin C (ascorbic acid), a water-soluble vitamin, exerts both antioxidant and non-antioxidant effects on the brain, including neurotransmitter synthesis. Its role in schizophrenia has been described in many preclinical and clinical studies mostly for its potent antioxidant activity and neuroprotection against oxidative stress induced by antipsychotic therapy. Vitamin C, as a cofactor of TET enzymes and histone demethylases, interferes with DNA demethylation and histone demethylation leading to epigenomic alterations. A recent clinical study explored the association between vitamin B and schizophrenia [96]. This population-based case-control study showed a correlation between lower pyridoxine levels and schizophrenia. Pyridoxine (vitamin B6) as its active form, pyridoxal-5'-phosphate, is a water-soluble vitamin that works as a co-factor for many enzymatic processes, and, in a recent preclinical study, pyridoxine increased the GAD67 expression in hippocampus suggesting a potential role in modulating GABAergic transmission [112, 113].

### 5.2 Essential Polyunsaturated Fatty Acids

Essential fatty acids (EFAs) such as the omega-3 polyunsaturated fatty acid (PUFA), α-linolenic acid (ALA) and its derivatives eicosapentaenoic acid (EPA) and docosahexaenoic acid (DHA) together with arachidonic acid (AA), a derivative from linoleic acid (LA), are dietary components that are crucial for brain structure and function, and PUFAs deficiency is considered a risk factor for schizophrenia [112, 114]. Dietary sources of ALA are green plants, nuts, flaxseed, and rapeseed oil, whereas oily fish is the main source of EPA and DHA. PUFAs are considered potent immunomodulators by triggering microglia cells, and changes in their brain content may cause abnormal neuroimmune interactions, including abnormalities in synapse elimination and plasticity, which are relevant during early developmental phases [115]. Microglia are brain innate immune cells that respond to noxious stimuli and inflammation with a production of pro- or anti-inflammatory factors in a crosstalk manner with astrocytes and also play a major in neurodevelopment and plasticity [116].

Omega-3 polyunsaturated fatty acids (PUFAs), such as α-linolenic acid (ALA), found mainly in seeds and oils, including flaxseed, walnuts, chia, hemp, and many common vegetable oils, and its derivatives EPA and DHA, show anti-inflammatory effects by regulating cytokine production, such as IL-6 or TNF-α and lowering leukocytes pro-inflammatory activity through NF-κB, which is responsible for proinflammatory genes expression whereas the saturated fatty acid palmitic acid through activation of Toll-Like Receptor (TLR) expressed on microglia cells induces a pro-inflammatory response *via* cytokine release, which in turn activate local astrocytes and promotes inflammatory response [117].

Dietary PUFAs directly modulate transcription factors involved in inflammation, most importantly PPAR-α/γ, NF-κB or TLR4 [118]. The anti-inflammatory effects of omega-3 fatty acids may be responsible for reducing psychosis severity or cognitive impairment in the high-risk population [119].

PUFAs such as EPA and DHA inhibit the production of proinflammatory cytokines through their interaction with the G protein-coupled receptor (GPR120) that leads to inhibition of NF-κB cascade or through their inclusion into the membrane lipid bilayer with consequent disruption of the TLR-4 signalling and activation of the IKKβ/NF-κB pathway, thus preventing cytokines formation [120]. Interestingly, PUFA deficiency during early neurodevelopment is also associated with epigenetic changes targeting the expression of PPAR-α [121]. Deprivation of PUFAs caused hypermethylation of Rxr and Ppar gene promoters associated with decreased expression in schizophrenia mouse models and humans, suggesting they can be considered a potential biomarker to identify a subgroup of the schizophrenia population. Moreover, exposure to PUFAs reversed the prenatal immune activation-induced global DNA hypomethylation in adult offspring [122].

### 5.3 Flavonoids

Natural bioflavonoids, including malvidin, quercetin, apigenin, luteolin and baicalin, are polyphenolic phytonutrients extensively studied for their antioxidant and anti-inflammatory activities and their potential application in chronic diseases, including schizophrenia [123]. For example, malvidin, found mostly in blueberries, represents one of the most largely diffuse anthocyanidins, which exhibits a strong antioxidant and anti-inflammatory activity and decreases inflammatory gene expression by inhibiting NF-κB signalling pathway and suppressing pro-inflammatory cytokine [124]. In addition, Malvidin-3′-O-glucoside (Mal-gluc), a malvidin metabolite, exerts its effects *via* epigenetic mechanisms by inhibiting histone deacetylase (HDAC)2 and modulates brain synaptic plasticity and peripheral inflammation [125]. Quercetin is a powerful antioxidant and anti-inflammatory flavonoid found in several foods, such as capers, goji berries, apples, and onions. It shows a wide range of biological *in vitro* and *in vivo* actions, including anti-carcinogenic, anti-inflammatory, antiviral activities, attenuation of lipid peroxidation and neuroinflammation by modulating the expression and function of cyclooxygenase, lipoxygenase, and production of interleukins [126]. Apigenin and luteolin, bioactive plant flavones found in celery, parsley, artichokes, and chamomile, show anti-inflammatory effects and protective effects against neurodegeneration and cognitive impairment by modulating epigenetic mechanisms, such as histone acetylation, activating BDNF signalling and decreasing neuroinflammation [127]. Luteolin showed several beneficial effects, including antioxidant, anti-inflammatory, microglia inhibition, neuroprotection, and memory increase [128]. Bioflavonoids exert their antioxidant and anti-inflammatory effects by regulating SIRT1/NF-κB signalling. Activation of SIRT1 inhibits NF-κB signalling through acetylation mechanisms that lead to inflammation resolution. Nutrition constitutes an epigenetic factor that modulates DNA methylation or histone acetylation [129]. For instance, sirtuins (SIRT1-7) are a class of HDAC, which play several physiological roles, including anti-inflammatory effects *via* NF-κB gene transcription inhibition, thus providing an epigenetic mechanism consistent with chromatin remodelling that enhances DNA transcription. For example, Sulphoraphane, an isothiocyanate in broccoli sprouts, and diallyl disulfide in garlic act as HDAC inhibitors [130]. Pharmacological regulation of HDAC activity induced by nutritional products, such as grape seed procyanidin, shows anti-inflammatory effects *via* secondary PPAR-α regulation in experimental animal models [131].

Catechins such as epicatechin gallate and epigallocatechin gallate (EGCG) are natural polyphenolic phytochemicals found largely in green tea and provide antioxidant effects due to their free radical scavenging ability. EGCG significantly inhibited high-fat diet-induced obesity and attenuated hypothalamic inflammation and microglia overactivation by regulating the NF-κB and STAT3 signalling pathways in mice [132]. Recent studies showed anti-inflammatory effects induced by EGCG by improving the high-fat- and high-fructose-induced cognitive defects through NF-κB pathway inhibition and by reducing human plasma lipid peroxidation caused by haloperidol, a first-generation antipsychotic [133].

Rutin, a biochemical compound found largely in buck-unpeeled apples and figs, shows anti-inflammatory, antioxidant, neuroprotective, nephroprotective, and hepatoprotective effects. Due to its antioxidant effects, rutin may benefit neurodegenerative disorders, including schizophrenia, by counteracting oxidative stress [134, 135].

Resveratrol, a polyphenolic phytoalexin found in grapes, and exerts several physiological activities, such as anti-aging, chemo-preventive, anti-carcinogenic, anti-inflammatory and antioxidant effects [136]. It has been extensively studied in several chronic diseases, ethanol-induced neuroinflammation and neurodegenerative diseases, including Alzheimer’s disease and schizophrenia, as shown in a recent clinical study [136]. Resveratrol supplementation improved metabolic status in patients with type-2 diabetes and coronary heart disease and upregulated PPAR-γ and SIRT1 [137].

### 5.4 Phytoestrogens

Recent evidence shows that the phytoestrogens, the isoflavones, daidzein and genistein down-regulate the expression of pro-inflammatory cytokines, such as COX-2 and iNOS, by activating PPAR and by inhibiting IκB activation [138].

They occur naturally and are found mostly in soybeans and soy products like tofu and exert antioxidant and neuroprotective activities. Studies examined the effects of daidzein on adipose-induced inflammation, causing insulin resistance in obesity. Jungbauer *et al.* (2014) showed that daidzein regulates adipokine expression through PPAR-γ, thereby improving the adverse effects of adipose inflammation, such as insulin resistance in obesity, which is common in individuals with psychosis. Also, a recent study shows that genistein induced changes in gut microbiota, improving glucose metabolism and cognitive function in mice fed a high-fat diet [139, 140].

### 5.5 Curcumin

Curcumin has also been studied in several clinical trials as a cognitive enhancer in neurodegenerative disorders and has a potent anti-inflammatory property [141]. It is the most active component of turmeric, isolated from the rhizomes of *Curcuma longa* and known to exhibit various pleiotropic properties, including antioxidant, anti-inflammatory, anti-amyloidogenic, lipophilic and cognition/memory enhancing activity, which suggests potential neuroprotective effects. Recent clinical studies analyzed the effects of curcumin on BDNF levels in schizophrenia or the beneficial effects of add-on therapy combined with antipsychotic treatment in patients with chronic schizophrenia. Curcumin has potential benefits in cognition and amyloid-induced neuronal toxicity, and it has been investigated extensively in Alzheimer’s disease [142-144].

### 5.6 Fibers

Fibers represent another group of nutritive compounds relevant for cardiovascular disease, diabetes, cancer, mineral absorption in the intestinal tract and GI health and microbiota [145]. Regular consumption of foods rich in dietary fibers, such as legumes and leafy greens, improves overall health and prevents various conditions, such as diabetes, atherosclerosis, diverticulitis, and obesity. A diet rich in fibers is recommended to reduce inflammatory biomarkers, such as C-reactive protein (CRP) and IL-6, which are also involved in neuroinflammatory mechanisms and schizophrenia [146].

## CONCLUSION

Schizophrenia is a severe and complex syndrome that involves multiple pathogenetic mechanisms, including brain structure abnormalities, epigenetic changes, neurotransmitters alterations, brain-microbiome-immune axis disruption, peripheral and central inflammation, and perineuronal nets abnormalities and glial cell activation. Individuals with schizophrenia, mostly caused by their functional disability and lack of family or social support, often opt for unhealthy dietary habits, which contribute to poor clinical outcomes. Antipsychotic medications, mostly effective against positive symptoms, target mainly specific dopamine and serotonin receptors with the latest antipsychotic, lumateperone, which also acts as a glutamate modulator. Although effective against clinical manifestation, these drugs fail to target neuroinflammation or comorbidities, including metabolic syndrome and cognitive impairments. Thus, alternative therapeutic strategies targeting anti-inflammatory interventions must be considered, for example, by adopting a functional nutrition diet regimen in mental health communities or home-based by implementing psycho-educational programs. Bioactive compounds target several molecular components involved in oxidation and inflammation processes. Also, several bioactive compounds show epigenetic effects by modulating epigenetic marks, including histone acetylation and DNA methylation, which then contribute to regulating gene expression. In addition, the nuclear receptors, PPARs or PARPs may represent suitable candidates for drug therapy targeting inflammatory-induced conditions such as schizophrenia and are worth further pre- and clinical investigations. PEA, for example, might be considered a valid therapeutic strategy for add-on therapy. Although the use of bioflavonoids such as quercetin, resveratrol, EGCG, vitamins, and minerals as supplements should be further investigated in clinical studies, adopting a functional anti-inflammatory diet rich in natural bioactive compounds may benefit the disease course and severity, reduce the risk of comorbidities, improve cognition and quality of life.

## HIGHLIGHTS

• Individuals affected by chronic schizophrenia show poor general health, poor nutrition, and a higher rate of metabolic syndrome and cardiovascular disorders leading to premature death.

• Immune-inflammatory mechanisms play a significant role in both schizophrenia progressiveness and treatment response, such as drug-resistance and metabolic side effects.

• The life expectancy of individuals with schizophrenia is lower than the general population's average due to unhealthy lifestyles associated with increased risk of cardiovascular diseases, diabetes, medication-induced metabolic syndrome, tobacco or alcohol use, and drug addiction.

• An integrated functional nutrition intervention is needed in combination with common treatments to modify the disease progression and increase life expectancy by targeting the risk of comorbidities.

• Functional foods with intrinsic antioxidant and anti-inflammatory activities modulate peripheral and brain inflammation, consequently improving overall health.

## Figures and Tables

**Table 1:** Bioactive compounds with antioxidant and anti-inflammatory properties.

**Phytochemical**	**Bioactive Compound**	**Food Source**	**Physiological Effects**
Anthocyanidins	*Malvidin*	Blueberries, black grapes, pomegranates	Antioxidant and anti-inflammatory activity.
Flavonols	*Kaempferol, myricetin, quercetin*	Berries, kale, grapes, spinach, bell peppers, cocoa, broccoli, sweet potatoes, tomatoes	Anti-carcinogenic, anti-inflammatory. antioxidant and antiviral activities. mitigation of microglia-mediated neuroinflammation.
Flavanones	Hesperidin, naringenin	Citrus fruits (lemons and oranges), grapes	Antioxidant, anti-inflammatory.
Flavones	Apigenin, luteolin	Celery, fresh parsley, olives, oregano, peppers, and rosemary	Suppression of oxidative stress *via* anti-inflammatory effects on NF-kB, brain support, protection and memory increase.
Flavanols	Epicatechin-gallates, procyanidins, catechin	Tea, grapes, lentils, cocoa, apples with peel on, apricots, cherries, peaches, blackberries, black grapes, strawberries, blueberries, and raspberries	Antioxidant. Free radicals’ scavengers. Decrease inflammation and microglia overactivation. Improve cognition.
Isoflavones	*Daidzein*	Grape seeds, soy products	Improve in adipose inflammation, and insulin resistance. Improve in cognitive function.
Flavans	*Genistein*	Soybeans	Antioxidant and neuroprotective activities. Improve in glucose metabolism, and cognitive function.
Flavonoid glycoside	*Rutin*	Buckwheat, apples with skin, asparagus (specially the bottom part), grapefruit, lemons, orange juice, oranges	Anti-inflammatory, antioxidant, neuroprotective, nephroprotective, hepatoprotective effects.
Phenolic acids	*Caffeic acid, ferulic acid*	Apples, coffee beans, blueberries, oranges, peaches, potatoes, pears	Antioxidant and anti-inflammatory properties. Improve cognition and neurodegeneration.
Hydroxy-benzoioc acids	*Gallic acids*	Grape and raspberry grape juice, longan seeds, strawberries	NF-κB inhibitors, anti-inflammatory properties.
Trihydroxy-stilbenes	*Resveratrol*	Grape skin, peanuts, red wine, cranberries	Anti-aging, chemo-preventive, anti-carcinogenic, anti-inflammatory and antioxidant effects.
Tannins/ Proanthocyanidins	*Catechin, tannic acids*	Coffee, cocoa, lentils, peas, walnuts, berries, olives, plums, tea, chickpeas, herbs and spices	Antioxidant properties.

**Table 2:** Effects of bioactive compounds in schizophrenia: clinical evidence.

**Bioactive Compound**	**Clinical Effect**	**Physiological Mechanism**	**References**
Omega-3 Fatty Acids	Improvement in cognitive dysfunction.	Increase in BDNF, Decrease in CRP, IL-6, TNFα	*Tang et al., 2020 Omega-3 fatty acids ameliorate cognitive dysfunction in schizophrenia patients with metabolic syndrome* [147]
Omega-3 Fatty Acids	Decreased inflammation levels in schizophrenia patients with metabolic syndrome.	Triglyceride metabolism; decreased TNF-alpha levels	*Xu et al., 2019 Effects of omega-3 fatty acids on metabolic syndrome in patients with schizophrenia: a 12-week randomized placebo-controlled trial* [148]
Vitamin D	Beneficial effects on PANSS score, and metabolic profiles.	Increased total antioxidant capacity; decreased hsCRP levels	*Ghaderi et al., 2019 Clinical and metabolic response to vitamin D plus probiotic in schizophrenia patients* [149]
Resveratrol	Greater improvement in negative, general psychopathology, and total scores.	Efficacy and tolerability of resveratrol add-on therapy in the treatment of negative symptoms in patients with stable schizophrenia	*Samaei et al., 2020 Resveratrol Adjunct Therapy for Negative Symptoms in Patients With Stable Schizophrenia: A Double-Blind, Randomized Placebo-Controlled Trial* [137]
Resveratrol	Metabolic syndrome.	Resveratrol supplementation improved total cholesterol (TC), High-density Lipoprotein cholesterol (HDL-c), Very-low density Lipoprotein cholesterol (VLDL-c), urea, creatinine and albumin serum levels	*Batista-Jorge et al., 2020 Oral resveratrol supplementation improves Metabolic Syndrome features in obese patients submitted to a lifestyle-changing program* [150]
Curcumin	Significant positive changes in both groups from baseline in all scales of measurement.	3000 mg/d curcumin or placebo combined with antipsychotics. The outcome measures were the Positive and Negative Symptoms Scale (PANSS) and the Calgary Depression Scale for Schizophrenia.	*Miodownik et al., 2019 Curcumin as Add-On to Antipsychotic Treatment in Patients With Chronic Schizophrenia: A Randomized, Double-Blind, Placebo-Controlled Study* [142]
Curcumin	Metabolic syndrome.	Increased weight loss; reduction of body fat; enhanced reduction of BMI	*Di Pierro et al., 2015 Potential role of bioavailable curcumin in weight loss and omental adipose tissue decrease: preliminary data of a randomized, controlled trial in overweight people with metabolic syndrome. Preliminary study* [151]
PPAR-α ligand (N-palmitoylethanolamide)	Role of plasma PEA in metabolic improvement.	Positive relationship between PEA and waist circumference, and metabolic parameters	*Pataki et al., 2018 Effects of a Weight Loss Program on Metabolic Syndrome, Eating Disorders and Psychological Outcomes: Mediation by Endocannabinoids?* [152]
Grape polyphenols	Metabolic syndrome.	Improvements in vascular function and reduce blood pressure.	*Barona et al., 2012 Grape polyphenols reduce blood pressure and increase flow-mediated vasodilation in men with metabolic syndrome* [153]

## LIST OF ABBREVIATIONS

AD: Alzheimer’s Disease
BDNF: Brain-derived Neurotrophic Factor
COPD: Chronic Obstructive Pulmonary Disease
CRP: C-reactive Protein
DNMT: DNA Methyltransferases
FGAs: First-generation Antipsychotics
GAD: Glutamate Decarboxylase
PD: Parkinson’s Disease
SGAs: Second-generation Antipsychotics
